# Retraction: *miRNA-148b* regulates radioresistance in non-small lung cancer cells via regulation of MutL homologue 1

**DOI:** 10.1042/BSR-20150300_RET

**Published:** 2021-04-22

**Keywords:** lung cancer, miR-148b, MutL homologue 1 (MLH1), radioresistance

This article is being retracted from *Bioscience Reports* at the request of the Editor in Chief and Editorial Board following a notification from the listed corresponding author, Jianbin Li, via an email address that differed to the one included with the submission. Jianbin Li confirmed that he was unaware of the submission and publication of the article, and that he did not conduct any part of this research, hence should not have been listed as an author on this paper.

The corresponding author listed on the paper as Jianbin Li, using email address 18678186986@163.com, later contacted the Editorial Office to request that the institution “Department of Radiotherapy, The Tumor Hospital of Shandong Province” was removed from the affiliations and from the paper, as well as to request that the corresponding author was changed from Jianbin Li to the first author, Guangsheng Zhai.

Following queries from the Editorial Office, the listed corresponding author (via the email address listed in the article), revoked the correction request and asked that the article was retracted, as they had been unable to replicate the results of the study.

In light of the concerns raised regarding the authorship of the paper, the unexplained request to remove all mentions of an institution and the inability to replicate the results, the Editorial Board is retracting the paper.

